# BEYOND HOUSING

**DOI:** 10.1093/geroni/igac059.2634

**Published:** 2022-12-20

**Authors:** Marianne Raimondo

**Affiliations:** Rhode Island College, Greenville, Rhode Island, United States

## Abstract

Social isolation and loneliness in older adults are a public health challenge that has been exacerbated during the COVID pandemic. While quarantining although it was necessary to protect individuals from the virus, older adults lost their usual social supports, connectedness, and relationships. Research has shown that loneliness and social isolation have many negative effects on a person’s physical and mental health. This presentation will describe an initiative of Age Friendly RI which addressed the problem of social isolation among older adults living in a congregate housing community who struggled with stress, anxiety, depression, and exacerbation of mental illness. Through a partnership between a state college and a community based mental health center, and supported with grant funding, behavioral health services and supports were embedded in the housing community. The project was multi-faceted involving community health workers and a community support professional who provided outreach and emotional support, the use of technology to help residents connect with medical providers or loved ones or enjoy music, games, movies, and students who provided outreach and taught older adults how to use technology. A social isolation scale was administered to identify residents at high risk of social isolation and targeted intervention. The evaluation of the program will be discussed in terms of the interventions implemented and the outcomes realized, including resident well-being and satisfaction. Finally, it will suggest a pathway for sustaining the integration of behavioral health services in housing to better meet the behavioral health needs of older residents beyond the pandemic.

